# Awareness, Experiences, and Attitudes Toward Preprints Among Medical Academics: Convergent Mixed Methods Study

**DOI:** 10.2196/78139

**Published:** 2026-04-17

**Authors:** Mustafa Sevim, Burak Karamese, Zafer Alparslan

**Affiliations:** 1 Department of Physiology, School of Medicine, Marmara University, Başıbüyük Yolu No: 9 D:2, Istanbul, 34854, Turkey, 90 2167775500; 2 School of Medicine, Marmara University, İstanbul, Turkey

**Keywords:** preprint, medical academics, publishing attitudes, editorial policies, survey

## Abstract

**Background:**

Preprints—scientific manuscripts shared publicly prior to formal peer review—are gaining momentum across academic disciplines. However, their adoption in clinical and biomedical sciences remains limited, particularly in countries where traditional publishing norms prevail. Editorial ambiguity and a lack of national policy further complicate their use.

**Objective:**

This study aimed to assess the awareness, experiences, and attitudes of medical academics at Marmara University School of Medicine toward preprints and to explore the editorial landscape through both journal editor feedback and a review of journal-level preprint policies.

**Methods:**

A cross-sectional survey was conducted with 103 medical faculty members. The questionnaire included demographic questions, Likert scale items, and multiple-choice items assessing knowledge, familiarity, and attitudes toward preprints, as well as open-ended items to explore concerns. A “preprint test score” (0‐4) was developed to quantify objective knowledge. Subgroup analyses were conducted by age (<40 vs ≥40 y) and academic discipline (basic vs clinical sciences). Additionally, all responses to open-ended questions from journal editors and 118 biomedical journals were manually reviewed for their stated stance on preprints and article processing charges (APCs). A convergent mixed methods design was used, combining a structured survey, thematic analysis of open-ended responses and editorial feedback, and a document-based review of biomedical journal policies.

**Results:**

Only 42.9% (n=34) of participants reported familiarity with the concept of preprints, and 13% (n=10) had previously published on a preprint server. Misconceptions about ethics, peer review, and compatibility with journal policies were common. Subgroup analysis revealed that older participants scored higher on the “preprint test” (mean 2.20, SD 1.31 vs mean 1.97, SD 1.60) and had more experience with preprint publishing (1/40, 2.5% of younger participants; 7/29, 24.1% of older participants). Further, younger academics expressed less openness toward future use (n=7, 17.5% in the younger group; n=8, 27.6% in the older group). Clinical faculty were generally more hesitant than basic science faculty, although both groups raised concerns about the academic recognition of preprints. Editorial responses reflected a mix of cautious endorsement and skepticism. Among the 118 biomedical journals reviewed, most lacked clear preprint policies, while a small number either explicitly prohibited or permitted them.

**Conclusions:**

There is limited awareness and cautious engagement with preprints among medical academics and editors in Türkiye. Generational and discipline-based differences further influence knowledge and attitudes. The lack of clear editorial guidance from biomedical journals may reinforce academic uncertainty. Tailored educational initiatives, transparent journal policies, and institutional support will be essential to foster a more open and inclusive scientific publishing environment.

## Introduction

Preprints—manuscripts publicly shared prior to peer review—have transformed the pace and openness of scholarly communication. Widely adopted in fields such as physics and computer science for decades, preprints enable rapid dissemination, open peer commentary, and broader visibility of research findings [1]. In recent years, the biomedical community has increasingly engaged with preprint platforms, particularly during public health crises such as the COVID-19 pandemic. For instance, between June 2020 and June 2022, the US National Library of Medicine made more than 3300 National Institutes of Health–funded COVID-19 preprints accessible in PubMed Central, marking a pivotal shift toward preprint integration in mainstream biomedical publishing [2].

Despite this global momentum, the adoption of preprints in clinical and medical sciences remains uneven [3]. In countries like Türkiye, where academic evaluation systems and journal structures still emphasize traditional peer-reviewed publication, the concept and utility of preprints are often misunderstood or undervalued. Anecdotal observations suggest hesitancy among medical faculty, fuelled by concerns about plagiarism, duplication, and lack of recognition in academic promotion criteria.

Journal editors also play a crucial role in shaping scholarly norms. Editorial policies on preprints vary widely across journals: while some encourage their use, others either prohibit them or do not explicitly mention them at all [4-7]. The absence of a clear preprint policy creates uncertainty for authors and may contribute to low adoption, particularly among early-career researchers concerned about publication eligibility [8].

While international studies have explored general attitudes toward preprints [3,9-11], little is known about how these perspectives vary within academic subgroups. Factors such as career stage and departmental discipline may influence both knowledge and perception. For instance, basic science researchers are often more open to experimentation with publishing models, whereas clinical academics may prioritize peer-reviewed evidence with clear implications for practice [12]. Similarly, younger faculty may view preprints as tools for early visibility and career advancement, while more senior academics may adhere to traditional notions of scholarly validation and prestige.

To date, no systematic assessment has examined the knowledge, attitudes, and editorial perspectives regarding preprints within Türkiye’s medical academic community. To address this gap, this study used a mixed methods design, integrating three data sources: a cross-sectional survey of medical academics at Marmara University School of Medicine; a document-based review of preprint and article processing charge (APC) policies from Turkish biomedical journals; and a descriptive analysis of qualitative feedback from journal editors.

In addition to characterizing general patterns, we examine subgroup differences by age and academic discipline to uncover nuanced barriers and opportunities for preprint adoption in the evolving landscape of scientific communication.

## Methods

### Overview

This study involved a cross-sectional survey design conducted at Marmara University School of Medicine in İstanbul, open-ended questions directed at journal editors, and document-based content analysis (convergent mixed methods). The goal was to evaluate the awareness, knowledge, and attitudes of medical academics toward preprints and to explore perceived barriers to preprint use.

Overall, this study harnessed three different data sources to evaluate this preprint issue:

- An online survey for medical academics at the Marmara University School of Medicine that included both quantitative and qualitative (open-ended questions) elements.
- A comprehensive review of editorial perspectives and policies of Turkish biomedical journals regarding preprints.
- Open-ended answers obtained from the editors of those biomedical journals regarding preprint policies and attitudes.

### Participant Recruitment and Data Collection

A structured online survey (closed survey) was created using SurveyMonkey to control all technical issues and distributed via institutional email lists and internal professional networks between April and July 2024. At the beginning of the survey, participants were informed about the survey and informed consent was obtained. The survey targeted medical academics from a variety of departments and academic ranks at Marmara University School of Medicine. We reached out to all 1529 medical academics. No personal identifiers were collected, and all responses were anonymized and aggregated for analysis.

Although the total number of participants was 108, not all respondents answered every question. Some skipped certain items, particularly in the later sections of the survey. As a result, the number of responses varies across different variables, and this is reflected in the sample sizes reported in the Results section.

### Survey Instrument

The survey included demographic items (eg, age, academic title, department), multiple-choice questions, and Likert scale questions assessing familiarity with preprints, previous use, attitudes toward preprints and peer review, and expectations for scientific quality and open-ended questions capturing perceived barriers and concerns related to preprint use.

To quantify objective knowledge of preprints, a “preprint test score” was generated from 4 multiple-choice questions. Participants received one point for each correct response, resulting in a total score ranging from 0 to 4. A higher score indicated higher knowledge of preprints. The responses to the relevant part of the survey (Question 9: “Tick the option you think is correct”) were used to calculate the preprint test score. The whole survey form can be found in Multimedia Appendix 1.

### Subgroup Analyses

To explore differences in preprint engagement and perceptions, participants were stratified into subgroups based on the following:

- Age: Participants were divided into two groups based on age—those younger than 40 years and those 40 or older. This age threshold was chosen for two primary reasons. First, it closely reflects the central tendency of our sample’s age distribution (mean 39.56, median 36, range 23‐73 years). Second, within the Turkish academic context, 40 represents a significant career milestone, often coinciding with the transition to an assistant professorship.
- Academic discipline: Basic sciences (eg, physiology, microbiology) versus clinical sciences (eg, internal medicine, surgery).

Subgroup comparisons were made for knowledge scores, attitudes, and future intent to use preprints, allowing the identification of generational and discipline-based trends.

### Editorial Perspectives From Turkish Biomedical Journals

To gather complementary insights into institutional attitudes toward preprints, we reviewed all journals indexed in the Web of Science (InCites dataset and Emerging Sources Citation Index), filtered for Türkiye as the country of publication and covering the time period from 2019 to 2023. From an initial list of 280 journals, 264 remained after excluding duplicates, inaccessible websites, and journals with unclear policies. The 2-year impact factors and Journal Citation Index (JCI) quartiles were obtained as well.

These journals were manually categorized into biomedical and nonbiomedical fields based on their scope and published content according to Web of Science categories. Journals within the disciplines of medical sciences, pharmacology, biology, veterinary sciences, and nursing were classified as biomedical. Based on this classification, we identified 118 biomedical journals indexed in the dataset and based in Türkiye (as of April 2025) (Multimedia Appendix 2).

Editors of these journals were contacted via email and invited to respond to three open-ended questions (Multimedia Appendix 3): (1) their journal’s current stance on preprints, (2) the rationale behind that stance, and (3) their views on the future role of preprints in academic publishing.

The email was sent to the editors-in-chief of all biomedical journals, and a total of 7 editors responded. Given the limited number of responses, the data were summarized descriptively rather than subjected to formal thematic analysis.

### Journal Policy Review

In parallel, the same 118 biomedical journals were reviewed to assess their formal policies on preprints and APCs. Policy information was manually collected by examining publicly available sections of the journals’ websites, including “Instructions for Authors,” “Editorial Policy,” and “Ethical Guidelines.”

This assessment was conducted in two rounds—first in February 2024 and again in April 2025—to track any changes in policy over time.

Journals were categorized as follows:

- Preprint policy: allowed, prohibited, or not mentioned.
- APC policy: obligatory, free, or case-by-case (where charges depend on article type or other conditions).

This analysis provided insight into the editorial infrastructure surrounding preprints in Türkiye and helped contextualize how journal-level policies may influence researchers’ behavior and perceptions.

### Study Design and Data Analyses

This study used a convergent mixed methods design integrating quantitative survey data, qualitative insights, and document-based content analysis to explore medical academics’ awareness and attitudes regarding preprints.

A cross-sectional online questionnaire included demographic questions, Likert scale questions, and multiple-choice items. Descriptive statistics (frequencies, percentages, means, medians, SDs) were used, and no inferential statistical testing was conducted. This survey was reported in accordance with the Checklist for Reporting Results of Internet E-Surveys (CHERRIES) [13] (Checklist 1).

Quantitative comparisons of journal policies were made using frequency counts and visualized via bar plots and heatmaps, with reference to impact metrics (eg, JCI quartiles).

Open-ended responses within the survey were analyzed using pattern-based thematic analysis. Commonly expressed concerns were coded inductively to identify recurrent barriers and perceptions regarding preprint use. Responses were grouped into themes such as plagiarism concerns, lack of academic recognition, policy confusion, and ethical ambiguity.

Editorial perspectives were obtained through open-ended email queries sent to biomedical journal editors. These responses were descriptively summarized to illustrate common institutional views and infrastructure limitations regarding preprint adoption.

Findings from the three data sources were integrated during interpretation to identify convergence and divergence. Quantitative trends were contextualized with qualitative themes and policy landscape shifts, enabling a holistic understanding of both individual attitudes and institutional structures shaping preprint practices in Türkiye.

### Ethical Considerations

The study was approved by the Marmara University Faculty of Medicine Non-Drug and Medical Device Research Ethics Committee (protocol code 09.2024.600; May 17, 2024). Informed consent was obtained from all participants prior to enrollment. To ensure participant confidentiality, all datasets were thoroughly de-identified for preceding statistical evaluation, and the study was conducted in accordance with the principles of the Declaration of Helsinki. No compensation was provided to participants.

## Results

### Journal Policy Review

As part of our review of Turkish biomedical journal policies, we analyzed the trends in preprint and APC policies at two time points: February 2024 and April 2025. This dual time point approach aimed to assess how Turkish biomedical journals are evolving in response to global shifts in open science practices and publication economics.

### Preprint Policies

Preprint policies were categorized into three groups:

- Allowed: Journals explicitly welcome or permit submissions that were previously posted as preprints.
- Prohibited: Journals clearly disallow submissions that have appeared as preprints.
- Not mentioned: No reference to preprints could be found on the journal’s official website.

**Figure 1.**
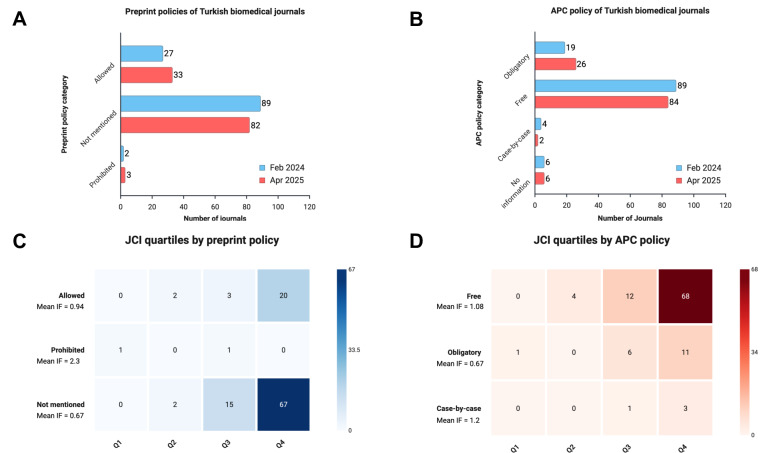
Preprint policy and article processing charge (APC) breakdown of Turkish biomedical journals. (A) Change in declared preprint policies of Turkish biomedical journals between February 2024 and April 2025. Journals that explicitly allowed preprints increased from 27 to 33, while those with no policy slightly decreased. (B) Change in APC policies of the same journals over the same period. A small rise in journals requiring obligatory APCs was observed. (C) Heatmap showing the distribution of journals across Journal Citation Index (JCI) quartiles by preprint policy, alongside mean journal impact factor (IF). Journals in all categories were predominantly concentrated in lower quartiles, particularly Q4; the prohibited group had the highest mean IF, but this category included only a very small number of journals. (D) Heatmap showing JCI quartile distribution by APC policy. Journals with case-by-case APCs had the highest mean IF, followed by free journals, whereas obligatory APC journals had a lower mean IF; all APC policy groups were concentrated mainly in Q3 and Q4.

Although the majority of journals still do not provide explicit guidance on preprints, our comparison over time revealed a modest but meaningful shift toward acceptance. Between February 2024 and April 2025, the number of journals explicitly allowing preprints increased from 27 to 33, while those with no policy decreased from 89 to 82. One additional journal began explicitly prohibiting preprints, increasing that category from 2 to 3 journals. These findings suggest a gradual trend toward policy transparency and a slow but positive normalization of preprint culture among Turkish biomedical journals (Figure 1A).

The implications of these policy differences are further reflected in journal performance metrics. As shown in the heatmap (Figure 1C), journals that allow preprints had a mean impact factor of 0.94, compared with 0.67 for journals that did not mention a preprint policy, whereas the prohibited category had the highest mean impact factor (2.3), although this was based on a very small sample.

### APC Policies

We also examined APC policies, grouping them into the following categories:

- Obligatory: Journals that always charge a fee for publication.
- Free: No publication charges to authors.
- Case-by-case: Charges apply only under certain conditions (eg, article type, page length).
- No information: No public declaration of APC policy found.

Between the two assessment periods, we noted a slight increase in journals with obligatory APCs (from 19 to 26), accompanied by a minor decrease in “free” journals (from 89 to 84). This indicates that APCs are becoming more common among Turkish biomedical journals, potentially impacting submission decisions, especially for early-career or unfunded researchers (Figure 1B).

When examining journal performance relative to APC policy, journals with case-by-case APC models showed the highest mean impact factor (1.2), followed by free journals (mean IF=1.08), whereas journals with obligatory APCs had a lower mean IF (mean IF=0.67) (Figure 1D). This suggests that journals with structured APC policies may be more established or competitive in the academic publishing ecosystem.

### Results of the Survey

#### Overview

A total of 103 medical academics participated in the study. Among those who reported sex (n=98), 51% were female and 49% were male. Age distribution (mean 39.56, SD 12.64, median 36 years) ranged from early 20s to over 70, with a wide representation from junior researchers to senior professors. In terms of academic titles, professors (28.1%) and residents (29.2%) made up the largest groups (Table 1).

**Table 1.** Demography of the participants (N=103).

Characteristic and categories		Values
Gender, n (%)		
	Female	50 (51)
	Male	48 (49)
Age (years), mean (SD)		39.56 (12.64)
Academic title, n (%)		
	Professor	27 (28.1)
	Associate professor	8 (8.3)
	Assistant professor	15 (15.6)
	Lecturer	7 (7.3)
	Resident (medical specialty)	28 (29.2)
	Other	11 (11.5)
Department, n (%)		
	Basic medical sciences	29 (30.2)
	Internal medical sciences	54 (56.3)
	Surgical medical sciences	13 (13.5)
	General practitioner	0 (0)
Scientific publications (last 5 y), n (%)		
	0	15 (15.6)
	1‐5	37 (38.5)
	6‐10	18 (18.8)
	11‐15	7 (7.3)
	16‐20	5 (5.2)
	≥20	14 (14.6)

#### Awareness of Preprints

A substantial portion of participants lacked awareness of preprints. Only 43% (n=36/84) of respondents reported familiarity with the term (mean test score of 2.76), while 21.4% (n=18/84; mean test score of 1.0) had never heard of it. Finally, 36% (n=30/84) had heard of the term but were not familiar with preprints (mean test score of 1.85).

#### Knowledge of Preprints

To quantify participants’ objective knowledge of preprints, a “preprint test score” was calculated based on responses to 4 multiple-choice questions. Participants received 1 point for each correct answer, resulting in a total score ranging from 0 to 4.

Overall, the mean preprint test score across all respondents was moderate (mean 2.07), with variation seen across both age groups and academic disciplines. Participants who reported being familiar with the term “preprint” scored highest (mean 2.76), while those who had never heard of the term scored the lowest (mean 1.00), suggesting internal validity of the test score.

#### Experience With Preprints

**Figure 2.**
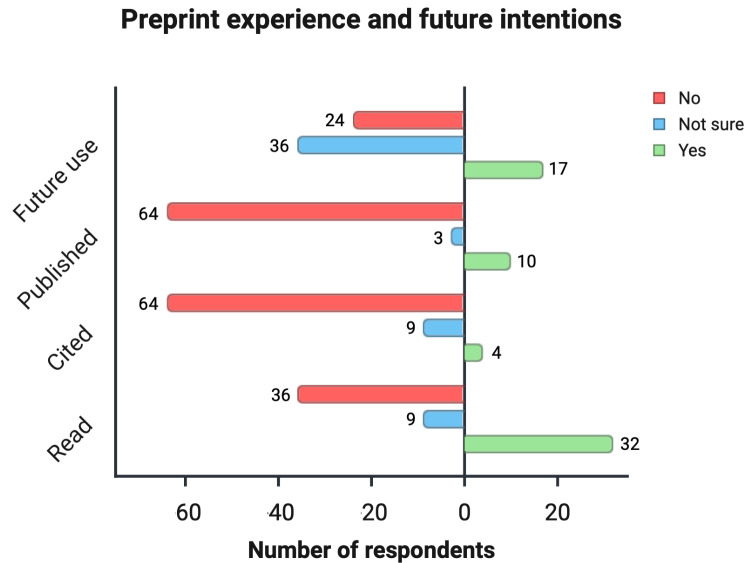
Experience with preprints among medical academics. Horizontal bar chart showing the distribution of responses regarding participants’ preprint-related behaviors and intentions, including whether they have read, cited, published, or plan future use of preprints. Responses are grouped by “yes,” “no,” and “not sure,” highlighting overall low levels of direct engagement with preprints.

Engagement with preprints was low across all indicators. Only 10 participants had published on a preprint server, 32 had read a preprint, and just 4 had cited a preprint in their scientific writing (Figure 2).

#### Attitudes Toward Future Use

When asked whether they would consider publishing a future manuscript as a preprint, only 22.1% (17/77) said yes, while 46.7% (n=36/77) were unsure and 31.2% (n=24/77) said no (Figure 2).

#### Subgroup Analyses

##### Subgroup Analysis by Age

Participants were categorized into two age groups: younger (<40 y, n=40) and older (≥40 y, n=29). The mean ages were 30.85 and 51.86 years, respectively. The older group demonstrated greater familiarity with preprints and higher preprint test scores (mean 2.20, SD 1.31 vs mean 1.97, SD 1.60). While only 2.5% (n=1) of younger participants had published a preprint, 24.1% (n=7) of older participants had done so. Further, younger participants showed less openness to future use, with 17.5% (n=7) considering preprint publication compared to 27.6% (n=8) in the older group, and a larger proportion remaining undecided (n=18, 62.5%).

Interestingly, younger participants expressed more favorable attitudes toward the role of preprints in scientific development and reforming the peer review system, whereas older participants valued conventional indicators of study quality such as citation counts and journal prestige. These differences are illustrated in Table 2.

**Table 2.** Attitudes and perceptions toward preprints by age group (N=69).^a^

Questions	<40 y (n=40)			≥40 y (n=29)		
	Agree	Neither agree nor disagree	Disagree	Agree	Neither agree nor disagree	Disagree
Preprints contribute to scientific development	29 (72.5)	11 (27.5)	0 (0)	13 (44.8)	12 (41.4)	4 (13.8)
The way preprints change the peer-review and editorial process is favorable for the future of science	20 (50)	18 (45)	2 (5)	9 (31)	13 (44.8)	7 (24.1)
A study loses its value if it is published only as a preprint	14 (35)	12 (30)	14 (35)	13 (44.8)	9 (31)	7 (24.2)
How much research parameters investigated in a study contribute to the value of the study	17 (42.5)	7 (17.5)	16 (40)	8 (27.5)	13 (44.8)	8 (27.5)
How many citations a study gets contributes to the value of the study	27 (67.5)	12 (30)	1 (2.5)	27 (93.1)	2 (6.9)	0 (0)
Which journal a study is published in contributes to the value of the study	29 (72.5)	8 (20)	3 (7.5)	25 (86.2)	3 (10.3)	1 (3.4)

a All values are n (%).

##### Subgroup Analysis by Academic Discipline

Participants were also grouped by academic department into basic sciences (n=24) and clinical sciences (n=45). The mean ages were 37.63 and 40.78 years, respectively. Preprint test scores and past use were similar across both groups. However, future intentions diverged: 29.2% (7/24) of basic science participants considered future preprint use, compared to 17.8% (8/45) in the clinical sciences group (data not shown). Additionally, 37.8% (17/45) of clinical faculty reported not considering preprint use in the future, suggesting a more cautious stance (data not shown). Attitudinal data further revealed that clinical scientists were more goal-oriented and focused on applicability in practice, whereas basic scientists placed more emphasis on the breadth of research parameters and openness to publication reform. Notably, more basic science participants believed that studies published only as preprints may lack value (n=14/24, 58.4%) compared to clinical scientists (n=13/45, 28.9%). These findings are visualized in Table 3.

**Table 3.** Attitudes and perceptions toward preprints by academic discipline (N=69).^a^

Questions	Basic sciences (n=24)			Clinical sciences (n=45)		
	Agree	Neither agree nor disagree	Disagree	Agree	Neither agree nor disagree	Disagree
Preprints contribute scientific development	16 (66.6)	8 (33.4)	0 (0)	26 (57.8)	15 (33.3)	4 (8.9)
The way preprints change the peer-review and editorial process is favorable for the future of science	13 (54.2)	10 (41.7)	1 (4.2)	16 (35.5)	21 (46.7)	8 (17.8)
A study loses its value of it is published only as a preprint	14 (58.4)	4 (16.7)	6 (25)	13 (28.9)	17 (37.8)	4 (8.9)
How much parameters investigated in a study contributes to the value of study	13 (54.2)	7 (29.2)	4 (16.6)	12 (26.7)	13 (28.9)	18 (44.4)
How much citation gets a study contributes to the value of study	17 (70.9)	6 (25)	1 (4.2)	37 (82.2)	8 (17.8)	0 (0)
Which journal a study published on contributes to the value of study	16 (66.6)	7 (29.2)	1 (4.2)	38 (84.4)	4 (8.9)	3 (6.6)

a All values are n (%).

### Barriers to Preprint Adoption

Understanding why academics and editors hesitate to engage with preprints is critical for developing targeted interventions. In this study, qualitative responses from survey participants were analyzed thematically and open-ended answers from editors were summarized descriptively to uncover common concerns.

#### Perceived Barriers Among Medical Academics (Survey Respondents)

Thematic analysis of open-ended responses revealed common concerns including fear of plagiarism or idea theft, lack of academic recognition, and insufficient knowledge about preprints. These barriers are summarized in Table 4.

**Table 4.** Common barriers to preprint use identified in participant comments (N=7).

Concern	Frequency (mentions)
Fear of plagiarism or idea theft	7
Preprints not valued in promotion	5
Lack of knowledge about preprints	6
Concerns about ethics or credibility	5
Journal policy restrictions	4

Participants were asked an open-ended question regarding their personal concerns or hesitations about using preprints. Thematic analysis revealed several recurring barriers:

- Fear of plagiarism or idea theft: A frequently mentioned concern was the potential for unreviewed ideas to be copied or republished without attribution. This concern appeared especially prominent among early-career researchers.
- Preprints not valued in promotion: Several participants indicated that preprints are not acknowledged in institutional promotion or academic evaluation processes. As a result, preprints were seen as a risky or unrewarding form of dissemination.
- Lack of knowledge about preprints: Many respondents were unsure about how to submit preprints, what platforms were reputable, or how preprints interact with formal journal submissions.
- Concerns about ethics or credibility: Some participants questioned whether preprints, by not undergoing peer review, could contribute to the spread of low-quality or misleading research.
- Journal policy restrictions: A few respondents mentioned that they avoided preprints because they believed many journals would reject submissions previously shared as preprints, even if that was not explicitly stated.

These findings suggest that barriers are shaped by both institutional norms and practical uncertainties.

#### Editorial Perspectives From Turkish Biomedical Journals

Responses from 7 journal editors in Türkiye revealed a spectrum of attitudes toward preprints, ranging from cautious support to open opposition.

One editor expressed support for preprints as a tool to promote transparency and protect authorship in a landscape where idea theft is perceived to be common. Some other editors described preprints as unnecessary for their journal, noting that they already publish accepted articles promptly and that their infrastructure does not support additional processing. Another editor viewed preprints skeptically due to the possibility of their misuse and the ethical complexity of assigning multiple DOIs to similar content. Another editorial opinion highlighted concerns over duplicate publication and the risk that preprints with assigned DOIs might be flagged as plagiarism in similarity checks. Despite these concerns, one editor predicted that preprints would become more widely accepted in the future, potentially reshaping the landscape of academic publishing in Türkiye.

Overall, the responses reflected uncertainty, infrastructural limitations, and a lack of standardization—factors that likely influence journal-level policies and affect how academics perceive the safety and legitimacy of preprint publishing.

## Discussion

Our findings reveal a notable gap in both awareness and practical engagement with preprints among medical academics at Marmara University School of Medicine. Despite the global momentum toward open science and rapid communication [14], many respondents exhibited limited familiarity with the concept, and a majority expressed hesitance or skepticism about its use. A recent global survey found that approximately 10% of medical and health sciences researchers in the United States were unfamiliar with preprints, and around 40% had never posted one [15]. In contrast, one-fifth of our participants had never heard of preprints, and four-fifths had never submitted one, highlighting a relatively greater degree of unawareness and hesitancy in our sample.

Misconceptions regarding peer review, duplicate publication, and ethical validity were widespread among respondents, underscoring the need for targeted education and clearer guidance. Maggio and Fleerackers [16] have proposed formally integrating preprint education into health professions curricula to improve understanding and normalize early dissemination practices. Moreover, research shows that students and early career researchers often struggle to distinguish preprints from peer-reviewed journal articles, reflecting a significant knowledge gap [17]. Consistent with these findings, our participants under the age of 40 demonstrated lower preprint knowledge, emphasizing the urgency of tailored interventions for this group.

Journal editors’ responses reflected a similar ambivalence. While some recognized the potential of preprints for visibility and transparency, others raised concerns about incompatibility with traditional editorial workflows, ethical ambiguity, and the potential for misuse. Despite such skepticism, studies have shown that peer-reviewed articles that were first posted as preprints tend to receive more citations and broader attention [18], suggesting tangible benefits to early sharing.

Our complementary policy analysis of 118 Turkish biomedical journals further highlights the cultural and structural barriers impeding broader preprint adoption. Only a small proportion of journals explicitly permitted preprints, while the vast majority either prohibited them or failed to mention them altogether. This lack of clear guidance stands in contrast to many high-ranking international clinical journals, which now explicitly allow or even encourage the submission of manuscripts previously posted as preprints [7]. The absence of formal policies among Turkish journals likely contributes to hesitation among authors, reinforcing an environment of academic conservatism and uncertainty—a trend previously noted in the literature [9].

However, our longitudinal review of journal policies between February 2024 and April 2025 suggests a slow but positive shift in this landscape. During this period, 6 additional journals formally adopted policies allowing preprints, while only 1 new prohibition was identified. Although incremental, this trend points toward a growing acceptance and normalization of preprint use within the Turkish biomedical publishing ecosystem. As journal policies become more explicit and aligned with global standards, uncertainty among researchers may diminish, potentially encouraging wider adoption of preprints in academic practice [19].

Additionally, variability in APC policies—particularly the prevalence of “free” and “case-by-case” models—may influence authors’ motivations. While one of the advantages of preprints is their cost-free accessibility, this incentive may be undercut if authors are already publishing in fee-free journals or are unaware of preprint benefits [20]. Financial considerations, combined with policy ambiguity, could thus create a disincentive for broader adoption.

Our subgroup analyses revealed how age and academic discipline influence perceptions of preprints. Older academics (≥40 y) were more likely to have published on preprint servers and scored higher on objective knowledge measures but also displayed more conservative attitudes, valuing journal prestige and citation counts. Younger academics (<40 y), on the other hand, were more pessimistic about the future potential of preprints, despite their limited experience. These generational differences may reflect an evolving academic culture that increasingly values transparency, speed, and accessibility.

This interpretation aligns with the findings of Fraser et al [20], who noted that junior researchers often use preprints to increase the visibility of their work, whereas senior researchers are more motivated by competitive concerns such as staking a priority claim [7].

An interesting finding is that, even though younger academics display reformist attitudes toward the conventional publishing process, they show less openness toward the use of preprints in future. This could be due to their relative inexperience or misconceptions regarding preprints, which can be validated by their lower preprint test score.

We also observed differences between basic and clinical science faculty members. While overall knowledge levels were similar, clinical faculty were more hesitant about preprint use. This may be due to the conservative nature of clinical research, which is closely tied to patient safety, regulatory compliance, and reliance on peer-reviewed evidence. As previously described, clinical medicine tends to be more cautious regarding the role of preprints in academic communication [21].

It is important to acknowledge that this study was conducted at a single academic institution in İstanbul. As such, our findings represent a localized snapshot and should be interpreted with caution. Additionally, while the survey captured a range of perspectives, the response rate was modest, and some questions had incomplete responses. The number of editor responses was also limited, restricting the depth of qualitative analysis. Finally, the policy review was limited to publicly available information, which may not fully reflect internal editorial practices or unpublished updates. Broader, multicenter studies will be necessary to determine whether these patterns hold across other regions and institutions in Türkiye.

To enable broader and more equitable adoption of preprints in the country, we recommend the following:

- Integration of preprints into academic evaluation and promotion criteria
- Development of clear and accessible editorial policies that explicitly state preprint compatibility
- Cross-disciplinary and interinstitutional dialogue to address differing perceptions of scientific quality and publishing ethics
- Encouragement of national or regional preprint platforms tailored to local academic contexts

Bridging both the awareness and policy gaps—and accommodating the diversity of views within academic institutions—is essential for aligning Türkiye’s scientific publishing culture with international standards.

This study identified a significant awareness gap and hesitancy toward preprints among medical academics at a Turkish university, driven by both individual misconceptions and a lack of clear, permissive policies from local biomedical journals. Despite a slow trend toward greater journal acceptance, attitudes remain divided by age and discipline. To foster a more open and timely research ecosystem, these findings underscore the need for strategic interventions, including targeted education to correct misconceptions, the establishment of transparent journal policies, and formal institutional recognition of preprints.

## Supplementary material

10.2196/78139 Multimedia Appendix 1 Survey form.

10.2196/78139 Multimedia Appendix 2 Preprint policies of Turkish biomedical journals.

10.2196/78139 Multimedia Appendix 3 Template email sent to editors-in-chief.

10.2196/78139 Checklist 1 CHERRIES checklist.

## Abbreviations

JCI: Journal Citation Index

## References

[1] Smart P (2022). The evolution, benefits, and challenges of preprints and their interaction with journals. Sci Ed.

[2] Funk K, Zayas-Cabán T, Beck J (2022). Phase 1 of the National Institutes of Health preprint pilot: testing the viability of making preprints discoverable in PubMed Central and PubMed. bioRxiv.

[3] Soderberg CK, Errington TM, Nosek BA (2020). Credibility of preprints: an interdisciplinary survey of researchers. R Soc Open Sci.

[4] Klebel T, Reichmann S, Polka J (2020). Peer review and preprint policies are unclear at most major journals. PLoS One.

[5] Choi YJ, Choi HW, Kim S (2021). Preprint acceptance policies of Asian academic society journals in 2020. Sci Ed.

[6] Teixeira da Silva JA, Dobránszki J (2019). Preprint policies among 14 academic publishers. The Journal of Academic Librarianship.

[7] Massey DS, Opare MA, Wallach JD, Ross JS, Krumholz HM (2020). Assessment of preprint policies of top-ranked clinical journals. JAMA Netw Open.

[8] Sarabipour S, Debat HJ, Emmott E, Burgess SJ, Schwessinger B, Hensel Z (2019). On the value of preprints: an early career researcher perspective. PLoS Biol.

[9] Ng JY, Chow V, Santoro LJ (2024). An international, cross-sectional survey of preprint attitudes among biomedical researchers. F1000Res.

[10] Baždarić K, Vrkić I, Arh E (2021). Attitudes and practices of open data, preprinting, and peer-review-a cross sectional study on Croatian scientists. PLoS One.

[11] Gutam S, Das S (2023). An overview of publication patterns in India’s agricultural research community: journals, open access, and preprints. Res Square.

[12] Pathak K, Marwaha JS, Chen HW, Krumholz HM, Matthews JB (2023). Open science practices in research published in surgical journals: a cross-sectional study. medRxiv.

[13] Eysenbach G (2004). Improving the quality of Web surveys: the Checklist for Reporting Results of Internet E-Surveys (CHERRIES). J Med Internet Res.

[14] Bertram MG, Sundin J, Roche DG, Sánchez-Tójar A, Thoré ESJ, Brodin T (2023). Open science. Curr Biol.

[15] Ni R, Waltman L (2024). To preprint or not to preprint: a global researcher survey. Asso for Info Science & Tech.

[16] Maggio LA, Fleerackers A (2023). Preprints in health professions education: raising awareness and shifting culture. Acad Med.

[17] Cataldo TT, Faniel IM, Buhler AG, Brannon B, Connaway LS, Putnam S (2023). Students’ perceptions of preprints discovered in Google: a window into recognition and evaluation. CRL.

[18] Fu DY, Hughey JJ (2019). Releasing a preprint is associated with more attention and citations for the peer-reviewed article. Elife.

[19] (2019). Springer Nature journals unify their policy to encourage preprint sharing. Nature New Biol.

[20] Fraser N, Mayr P, Peters I (2022). Motivations, concerns and selection biases when posting preprints: a survey of bioRxiv authors. PLoS One.

[21] Blatch-Jones AJ, Recio Saucedo A, Giddins B (2023). The use and acceptability of preprints in health and social care settings: a scoping review. PLoS ONE.

[22] BioRender BioRender.

