# Supplementary material for: Awareness, Experiences, and Attitudes Toward Preprints Among Medical Academics: Convergent Mixed Methods Study
Source: JMIRx Med. 2026 Apr 17;7:e78139. doi: 10.2196/78139 (PMC13093874; doi:10.2196/78139)
Supplement: Multimedia Appendix 1 [file xmed-v7-e78139-s001.pdf]

Preprint Awareness
Information and Consent Form

* 1. The purpose of this study, titled "Academics' Awareness and Opinions on Preprints in Turkey," is to measure participants' knowledge and awareness of preprints. The form takes an average of 6 minutes to complete.

No participant's name or contact information will be processed in the study, and your responses will be evaluated collectively, not individually.

Thank you in advance for your participation and responses.

◯ I have read and accept the "Information and Consent Form".

Preprint Awareness
Demographic Information

On this page you will be asked questions about your demographic information.

* 2. What is your sex?

◯ Male

◯ Female

* 3. How old are you ?

[ ]

Preprint Awareness
Academic Qualifications

4. Which one fits your status?

- ◯ Professor
- ◯ Associate professor
- ◯ Assistant professor
- ◯ Lecturer/Postdoc
- ◯ Subspecialist
- ◯ Clinical fellow
- ◯ Specialist physician
- ◯ PhD student
- ◯ Graduate student
- ◯ General practitioner
- ◯ Other (please specify)

5. Which department do you work in?

- ◯ Basic Medical Sciences
- ◯ Clinical Medical Sciences
- ◯ Surgical Medical Sciences
- ◯ General practitioner

6. How many scientific publications have you published in the last 5 years?

- ◯ 0
- ◯ 1-5
- ◯ 6-10
- ◯ 11-15
- ◯ 16-20
- ◯ 20+

7. What do you think makes a manuscript valuable?

	Totally disagree	Disagree	Neither disagree nor agree	Agree	Totally agree
The journal in which it was published	☐	☐	☐	☐	☐
How to handle the hypothesis	☐	☐	☐	☐	☐
How many citations has it received	☐	☐	☐	☐	☐
How many parameters are considered	☐	☐	☐	☐	☐
In which country was it made?	☐	☐	☐	☐	☐

Preprint Awareness

Preprint Familiarity

* 8. Have you heard of the concept of preprint before?

- ◯ Never heard of it
- ◯ Heard of it but not familiar with it.
- ◯ Heard and familiar with it

* 9. Tick the option you think is correct.

	True	False	Not sure
“In order for a preprint to be published, it is necessary to first submit it to a journal.”	◯	◯	◯
“For a preprint to be published, it must first be published in a journal or accepted by a journal.”	◯	◯	◯
“If a manuscript has been published as a preprint, it absolutely cannot be published in a journal.”	◯	◯	◯
"Preprints cannot be cited in scientific journals."	◯	◯	◯

* 10. Is there peer review on preprints?

◯ Yes

◯ No

◯ Not sure

11. Based on your answer, state what kind of evaluation you think it is or why you think it is not an evaluation.

* 12. Is there an editorial process for preprints?

- ◯ Yes
- ◯ No
- ◯ Not sure

13. Based on your answer, explain what kind of editorial process you think there is or why you think there is no editorial process.

[ ]

Preprint Awareness

Preprint usage practices

* 14. Have you ever published a manuscript on a preprint server?

- ◯ Yes
- ◯ No
- ◯ Not sure

15. Why did you choose to publish or not publish a manuscript on a preprint server?

[ ]

* 16. Have you ever read a preprint that you were not the author of?

- ◯ Yes
- ◯ No
- ◯ Not sure

17. Why did you choose to read or not read a preprint?

[ ]

* 18. Have you ever cited a preprint in any of your manuscript?

- ◯ Yes
- ◯ No
- ◯ Not sure

19. Why did you choose or not to cite a preprint?

[ ]

* 20. Are you considering publishing a manuscript as a preprint in the future?

- ◯ Yes
- ◯ No
- ◯ Not sure

21. Why do you consider or not consider publishing a manuscript as a preprint?

[ ]

* 22. Which preprint server/application/site have you used to publish your article before? (You can select more than one)

- [ ] MedRxiv
- [ ] BioRxiv
- [ ] ArXiv
- [ ] ScienceOpen
- [ ] Preprints.org
- [ ] ResearchHub
- [ ] ResearchGate
- [ ] ResearchSquare
- [ ] Not sure
- [ ] Never used it
- [ ] Other (please specify)

* 23. Which preprint server/application/site have you used to read an article before? (You can choose more than one)

- [ ] MedRxiv
- [ ] BioRxiv
- [ ] ArXiv
- [ ] ScienceOpen
- [ ] Preprints.org
- [ ] ResearchHub
- [ ] ResearchGate
- [ ] ResearchSquare
- [ ] Not sure
- [ ] Never used it
- [ ] Other (please specify)

* 24. What is the preprint policy of the journal you last applied to publish a manuscript in?

- ◯ Having a preprint of the manuscript prevents submission to the journal.
- ◯ Having a preprint of the manuscript does not prevent submission to the journal.
- ◯ It is mandatory to have a preprint of the manuscript.
- ◯ The journal has no policy regarding preprinting of the manuscript.
- ◯ I'm not sure if the journal has a policy regarding preprinting of the manuscript.
- ◯ I have not published a manuscript in a journal.

Preprint Awareness

Preprints and current journals

* 25. How much do you agree with the following opinions?

	Totally disagree	Disagree	Neither agree nor disagree	Agree	Totally agree
"I am satisfied with the peer-review and editorial processes of current scientific journals"	◯	◯	◯	◯	◯
"Paid journals have a negative impact on my accessibility to publications."	◯	◯	◯	◯	◯
"The progress of the current journal publishing system without major changes is negative for the future of science."	◯	◯	◯	◯	◯

* 26. How much do you agree with the following opinions?

	Totally disagree	Disagree	Neither disagree nor agree	Agree	Totally agree
"The way preprints are transforming the peer-review and editorial process is good for the future of science."	◯	◯	◯	◯	◯
"A valuable manuscript loses its value if it is published only as a preprint."	◯	◯	◯	◯	◯
"Preprints also receiving DOI numbers would harm the scientific publication process."	◯	◯	◯	◯	◯
"Preprints contribute to the development of scientific knowledge"	◯	◯	◯	◯	◯
"In the future there should only be preprints."	◯	◯	◯	◯	◯

27. What would you like to say about preprints in general?
