# Supplementary material for: Awareness, Experiences, and Attitudes Toward Preprints Among Medical Academics: Convergent Mixed Methods Study
Source: JMIRx Med. 2026 Apr 17;7:e78139. doi: 10.2196/78139 (PMC13093874; doi:10.2196/78139)
Supplement: Multimedia Appendix 3 [file xmed-v7-e78139-s003.pdf]

Dear Editor,

My name is Mustafa Sevim, who is lecturer on Marmara University School of Medicine Physiology Department. Me and my two friends from same university have been conducting a study named “Awareness, Experiences, and Attitudes Toward Preprints Among Medical Academics” with the ethical approval of Marmara University Faculty of Medicine Non-Drug and Medical Device Research Ethics Committee (protocol code 09.2024.600 and date of 17 May 2024) and we sent this e-mail to you regarding this study.

In order to enrich this study, we have reviewed journals whose origin in Türkiye indexed in Web of Science ESCI about their preprint policies.

As far as we have reviewed, your journal has no declared preprint policy
As far as we have reviewed, your journal allows submission of preprints
As far as we have reviewed, your journal prohibits submission of preprints

We consider that traditional, peer-reviewed, official journals are natural stakeholders of this preprint discussions ongoing. That’s why, we kindly request you to answer 3 questions below, thus adding your precious opinions on this discussion and study.

We guarantee that your answers will be evaluated anonymously and will never be published to 3rd parties publicly indicating the sources of answers. We hope you to share your ideas and contribute this discussion. We thank you for your time and consideration.

Best regards,

Dr. Mustafa Sevim
(Phone number and email address)

Questions:

1. Why does your journal have no declared preprint policy ?/ Why does your journal allow preprints ? / Why does your journal prohibits preprints ?

2. Why do other journals have no declared preprint policy, you think ? / Why do other journals allow preprint, you think ?/ Why do other journals prohibits preprints ?

3. What are your opinions regarding future of preprints ?
